# Dynamic Computed Tomography Findings as Indicators of Uterine Artery Embolization in Postpartum Hemorrhage

**DOI:** 10.1001/jamanetworkopen.2025.12209

**Published:** 2025-05-23

**Authors:** Munekage Yamaguchi, Akihito Sagara, Yasunori Nagayama, Jun Morinaga, Yoshinori Yamanouchi, Shintaro Makino, Shigetaka Matsunaga, Hiroaki Tanaka, Junichi Hasegawa, Tomoaki Ikeda, Takashi Sugiyama, Eiji Kondoh

**Affiliations:** 1 Department of Obstetrics and Gynecology, Faculty of Life Sciences, Kumamoto University, Kumamoto, Japan; 2 Department of Diagnostic Radiology, Faculty of Life Sciences, Kumamoto University, Kumamoto, Japan; 3 Department of Clinical Investigation, Kumamoto University Hospital, Kumamoto, Japan; 4 Department of Obstetrics and Gynecology, Juntendo University Faculty of Medicine, Tokyo, Japan; 5 Center for Maternal, Fetal and Neonatal Medicine, Saitama Medical Center, Saitama Medical University, Kawagoe, Japan; 6 Department of Obstetrics and Gynecology, Mie University Graduate School of Medicine, Tsu, Japan; 7 Department of Obstetrics and Gynecology, Kumamoto General Hospital, Japan Community Health Care Organization, Kumamoto, Japan; 8 Department of Obstetrics and Gynecology, St Marianna University School of Medicine, Kawasaki, Japan; 9 Department of Obstetrics and Gynecology, School of Medicine, Ehime University, Matsuyama, Japan

## Abstract

**Importance:**

Postpartum hemorrhage (PPH) remains the leading cause of maternal mortality globally. Although traditional management relies on blood loss quantification, identifying cases resistant to conventional treatment remains challenging, potentially delaying crucial interventions.

**Objective:**

To determine the prevalence and clinical implications of PPH that is resistant to treatment showing arterial contrast extravasation on dynamic computed tomography (CT) (PRACE) and its association with intervention requirements.

**Design, Setting, and Participants:**

A multicenter retrospective case-control study of PPH cases was conducted at 43 tertiary facilities across Japan, including 30 university hospitals, between January and December 2021. The study included patients with PPH who were either transported to the centers or who delivered there. Data analysis was performed from September 2023 to November 2024.

**Exposure:**

A diagnosis of PPH, defined as blood loss exceeding 2000 mL or requiring more than 10 units of red blood cell transfusion.

**Main Outcomes and Measures:**

The primary outcome was the prevalence of PRACE and its association with uterine artery embolization (UAE) requirement. Secondary outcomes included risk factors for severe coagulopathy (fibrinogen <150 mg/dL). Odds ratios (ORs) were estimated using multivariable logistic regression to evaluate the association between types of PPH (atonic uterus, tissue, and PRACE) and PPH severity.

**Results:**

Among 352 patients (median [IQR] age, 33.0 [30.0-37.0] years; 211 [60.0%] primiparous), 205 (58.2%) underwent CT scans, with PRACE detected in 58 (32.2%) of evaluable cases. Patients with PRACE had significantly higher total blood loss (median [IQR], 3455 [2000-5070] mL vs 2500 [1500-2650] mL) and greater UAE requirement (50 of 58 patients [86.2%] vs 35 of 122 patients [28.7%]) compared with patients without PRACE. PRACE was the primary factor associated with the need for UAE (OR, 27.74; 95% CI, 10.52-83.14).

**Conclusions and Relevance:**

In this retrospective case-control study of patients with severe PPH undergoing dynamic CT, PRACE represented a distinct and common pathology in severe PPH and was associated with the need for interventional procedures. These findings suggest that dynamic CT imaging should be considered as an essential diagnostic tool in managing treatment-resistant PPH cases.

## Key Points

**Question:**

What are the clinical implications and prevalence of postpartum hemorrhage (PPH) resistant to treatment showing arterial contrast extravasation on dynamic computed tomography (CT) (PRACE) in severe PPH cases?

**Findings:**

In this multicenter case-control study of 352 severe PPH cases from 43 institutions, PRACE was identified in 32.2% of patients who underwent dynamic CT imaging, and its presence was significantly associated with higher rates of uterine artery embolization (86.2% vs 28.7% in non-PRACE cases) and greater transfusion requirements.

**Meaning:**

These findings suggest that PRACE represents a distinct and common pathology in severe PPH that is associated with the need for interventional procedures.

## Teaser

This case-control study examines the prevalence and clinical implications of severe postpartum hemorrhage that is resistant to treatment and shows arterial contrast extravasation on dynamic computed tomography (CT).

## Introduction

Postpartum hemorrhage (PPH) remains a critical global health issue as the leading cause of maternal mortality, accounting for approximately 8% of maternal deaths even in developed regions.^1^ Prompt and effective management of PPH is crucial, as delays in intervention can markedly increase mortality risk. The primary cause of transfusion-requiring PPH is atonic uterus, followed by retained placental tissue, including cases of placenta accreta spectrum without previa.^2^ These conditions are challenging to identify before delivery and may resist standard interventions, such as uterotonics and balloon tamponade, thereby necessitating invasive procedures like uterine artery embolization (UAE) or hysterectomy.

Traditionally, PPH diagnosis is based on quantifying blood loss^3^; however, severe PPH that is resistant to standard treatment cannot always be identified solely through blood volume measurements. At present, no globally standardized, reliable diagnostic method exists to identify PPH cases that fail to respond to conventional treatment. In prior studies,^4,5,6^ we identified a subset of patients with severe PPH exhibiting contrast extravasation into the uterine cavity on early-phase dynamic computed tomography (CT) scans, many of whom required surgical interventions. To describe this condition, we introduced the term *PRACE* (postpartum hemorrhage, resistance to treatment, and arterial contrast extravasation). The single-center retrospective analysis of 30 patients with PPH who underwent dynamic CT scans suggested an association between PRACE and cases requiring UAE, whereas embolization was generally unnecessary in cases where PRACE was not observed.^5^ This finding indicates a potential shift in traditional PPH management strategies.

Since those initial studies, the use of dynamic CT in guiding PPH management has gained traction in Japan.^7,8^ However, PRACE remains a novel concept globally, lacking standardized terminology or definition. Although our initial study provided valuable insights, its small sample size and single-institution scope limited the generalizability of its findings. Hence, this study, conducted as part of the Japan Society of Obstetrics and Gynecology initiative, seeks to illuminate the prevalence and attributes of PRACE in Japan by incorporating a larger, multicenter dataset collected from maternal, fetal, and neonatal medicine centers across Japan, reinforcing the clinical importance of PRACE and its potential implications for optimizing PPH management strategies.

## Methods

### Study Design

This retrospective case-control study was conducted in accordance with the Strengthening the Reporting of Observational Studies in Epidemiology (STROBE) reporting guidelines for case-control studies.^9^ This study was conducted on PPH cases managed at 43 tertiary facilities across Japan, including 30 university hospitals, between January and December 2021. Approval for the study protocol was obtained from the institutional review board of Kumamoto University. Informed consent was not individually obtained because the study was retrospective and used deidentified data. Instead, an opt-out approach approved by the Ethics Committee of Kumamoto University was used, with administrative approval obtained at each participating institution.

### Setting and Participants

The study population comprised both patients with PPH transported to these centers and those who delivered at these facilities and experienced obstetric hemorrhage resulting in a blood loss of over 2000 mL^10^ or requiring more than 10 units of red blood cell (RBC) transfusion.^11,12^ Inclusion criteria encompassed cases diagnosed with PPH due to atonic uterus, retained placenta, and placenta accreta spectrum without previa. These PPH types often complicate timely intervention decisions, such as the need for UAE or hysterectomy. In contrast, PPH arising from sources outside the uterus (eg, vaginal or perineal hematomas) or structural issues such as uterine inversion and rupture have well-established treatment protocols. Therefore, exclusion criteria included placenta previa, placental abruption, hematomas in the vaginal wall, vulva, or perineal area, uterine inversion, uterine rupture, cervical laceration, and intraperitoneal bleeding.

### Outcomes and Variables

The primary end points of the study were the prevalence and characteristics of PRACE and the associations among the risk factors and the need for UAE. The secondary end points were risk variables associated with minimum fibrinogen levels less than 150 mg/dL. Exposures of interest included maternal age, pregnancy history, method of conception, presence of myoma or adenomyosis, gestational age at delivery, mode of delivery, cause of PPH, amount of blood loss, shock index, hemoglobin level, fibrinogen level, transfusion volume, utilization of dynamic CT scan, administration of fibrinogen, administration of tranexamic acid, balloon tamponade, and UAE.

### Data Sources and Measurement

#### Data Collection

Medical records from each center were reviewed to collect data on various variables for each case. The diagnosis of atonic uterus, retained placenta, and placenta accreta spectrum without previa was based on the clinical assessment by obstetricians at each facility. Owing to diagnostic limitations, some cases of retained placenta may have included placenta accreta spectrum without previa, but retrospective differentiation was not feasible. The indications for dynamic CT scans varied according to institutional protocols and obstetrician discretion. Some facilities did not perform dynamic CT scans, whereas others routinely conducted CT scans following initial PPH management. In addition, certain facilities selectively used CT imaging to identify bleeding sources in cases of refractory PPH unresponsive to primary interventions.

#### Image Evaluation and Classification of PPH Types

Dynamic CT images were independently evaluated by 3 examiners, consisting of 2 obstetricians (E.K. and M.Y.) and 1 radiologist (Y.N.), using a Picture Archiving and Communication system (Shade Quest/View R; Fujifilm Solution Co, Ltd). Any discrepancies in PRACE diagnoses were resolved through consensus among all 3 examiners. Cases with inadequate imaging conditions (eg, absence of contrast media or lack of imaging during the early phase) or evident bleeding from obstetric lacerations (eg, cervical lacerations or vaginal wall lacerations) were excluded from the analysis.

**Figure 1.**
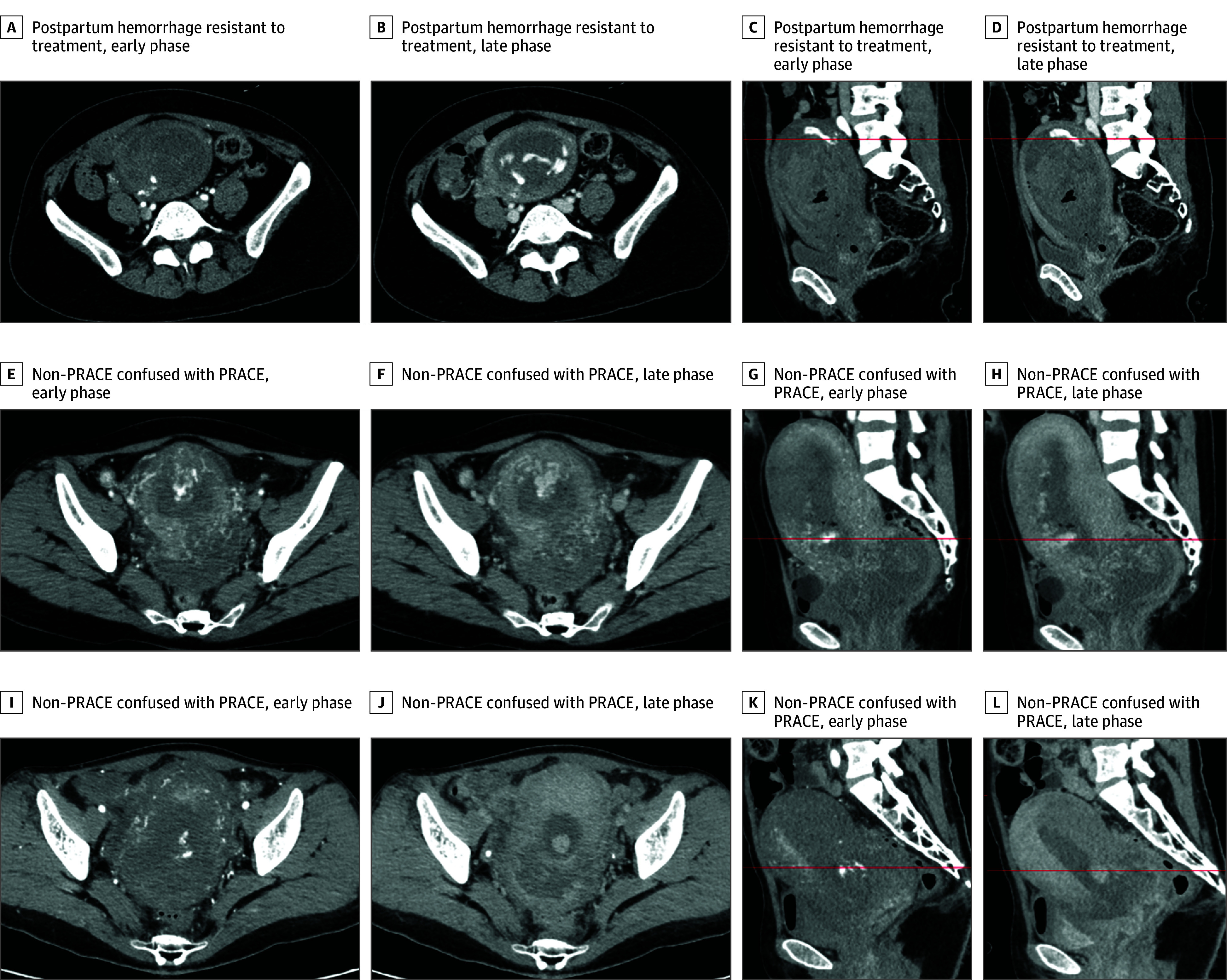
Evaluation of Dynamic Computed Tomography (CT) Images A-D, Representative case of postpartum hemorrhage resistant to treatment showing arterial contrast extravasation on dynamic CT (PRACE). Contrast media extravasation into the uterine cavity is observed in the early phase (A and C), with subsequent spread throughout the uterine cavity in the late phase (B and D). E-H, Instance of non-PRACE potentially confused with PRACE. Contrast extravasation into the uterine cavity appears prominent in the early phase, but no contrast diffusion is observed in the late phase (F and H). These images depict partial staining of the anterior uterine wall rather than active bleeding. I-L, Another illustration of non-PRACE with potential for misinterpretation as PRACE. Contrast leakage into the uterine cavity seems evident in the early phase. Subsequent phases reveal mild contrast spreading with well-defined borders, suggesting contrast uptake by the retained placenta. However, no contrast leakage into the uterine cavity is identified. These imaging findings suggest the presence of retained placenta without active hemorrhage. The red line in the sagittal section corresponds with the plane in the transverse section of each case.

**Figure 2.**
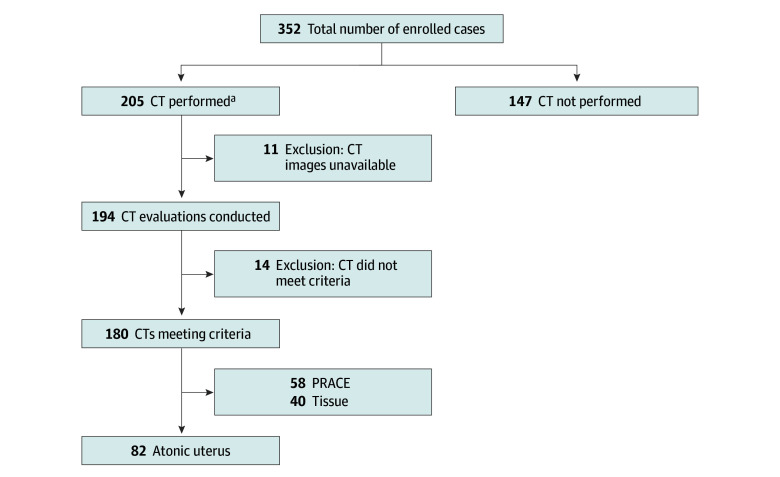
Flowchart of Case Selection and Classification of Postpartum Hemorrhage Types Based on Dynamic Computed Tomography (CT) Evaluation PRACE indicates postpartum hemorrhage resistant to treatment showing arterial contrast extravasation on dynamic CT. ^a^Indications for dynamic CT scans were determined according to institutional protocols and obstetrician discretion.

PRACE was defined as cases exhibiting early-phase contrast agent extravasation into the uterine cavity followed by subsequent spread in the late phase, regardless of the quantification of extravasation observed (Figures 1A to 1D). Cases without contrast agent leakage into the uterine cavity in the early phase (Figures 1E to 1H), or those showing contrast staining of the residual placental tissue in the uterus without subsequent contrast agent leakage into the uterine cavity in the late phase (Figures 1I to 1L), were classified as non-PRACE. Initial classification of PPH types was based on the attending physician’s assessment at each facility. Final diagnostic classification was determined by the authors through reanalysis of all CT images, prioritizing overlapping causes in the following order: (1) PRACE when arterial contrast extravasation was detected, (2) tissue in the presence of placental remnants, and (3) atonic uterus for all remaining cases (Figure 2). Each case was assigned a single PPH type according to this hierarchical classification to ensure mutually exclusive categorization.

### Bias

To minimize sampling bias, this study was conducted as a multicenter collaborative research effort to ensure that the study population reflects the characteristics of patients with PPH across Japan. To reduce information bias, standardized data collection procedures were implemented across institutions. Patient data were recorded in a unified format using a standardized Excel sheet (Microsoft) on a secure cloud-based platform, ensuring consistency across all participating centers. To mitigate selection bias, cases and controls were obtained from the same facilities to minimize differences in patient management. In addition, the classification of dynamic CT findings was conducted independently, with examiners blinded to clinical information, thereby reducing potential interpretation bias.

### Study Size

Given the retrospective case-control design, the sample size was not predetermined. The sample size was determined according to the available cases rather than a predefined calculation, ensuring that the analysis was conducted on all eligible cases. Since the study aimed to characterize PRACE rather than assess CT utilization patterns, the analysis was conducted on the basis of the presence or absence of PRACE.

### Statistical Analysis

Data analysis was performed from September 2023 to November 2024. Continuous data were expressed as median (IQR) in the descriptive analysis, and all group comparisons were performed using the Mann-Whitney *U* test. Univariable analyses were conducted to assess between-group differences. Categorical variables were evaluated using the χ^2^ test. Statistical significance was set at *P* < .05. *P* values were calculated with 2-sided tests. Odds ratios (ORs) and 95% CIs were estimated using multivariable logistic regression to evaluate the association between types of PPH (atonic uterus, tissue, and PRACE) and PPH severity, defined as the need for UAE and/or minimum fibrinogen levels less than 150 mg/dL. Two separate multivariable logistic regression models were used. The first assessed the association between UAE requirement and PPH types, adjusted for maternal age (≥35 years), fibroids or adenomyosis, labor analgesia, labor induction or augmentation, cesarean delivery, and minimum fibrinogen levels less than 150 mg/dL. The other model assessed the association between minimum fibrinogen levels less than 150 mg/dL and PPH types, adjusted for the same variables except for fibrinogen level. All statistical analyses were conducted using Prism statistical software version 10.2 (GraphPad Software).

## Results

A total of 352 patients were included in the study, and their characteristics are outlined in eTable 1 in Supplement 1. The median (IQR) maternal age was 33.0 (30.0-37.0) years, with 60.0% (211 of 352 patients) being primiparous. A total of 37.9% of patients (120 of 317 patients) conceived through in vitro fertilization, and fibroids or adenomyosis were present in 7.9% of cases (27 of 341 patients). Labor induction or augmentation was conducted in 45.3% of cases (154 of 340 patients), whereas operative vaginal delivery occurred in 22.2% of cases (78 of 352 patients) and cesarean delivery occurred in 26.4% of cases (93 of 352 patients). Atonic uterus was the leading cause of PPH in 73.3% of cases (258 of 352 patients), followed by retained placental tissue, including placenta accreta spectrum without previa and retained placenta, which accounted for 26.7% of cases (94 of 352 patients). The median (IQR) total blood loss was 2487 (1637-3420) mL in 327 patients, and minimum fibrinogen levels were below 150 mg/dL in 25.9% of cases (89 of 343 patients). Blood transfusions were administered to 77.3% of patients (272 of 352 patients), with a median (IQR) of 6 (2-10) units of RBCs and 4 (0-8) units of fresh frozen plasma. UAE was performed in 29.8% of cases (105 of 352 patients), whereas hysterectomy was required in 2.3% of cases (8 of 352 patients). Among the patients, 58.2% (205 of 352 patients) underwent CT scans, with PRACE detected in 58 evaluable cases (32.2%). A comparison of the clinical characteristics between the CT group and the non-CT group is shown in eTable 2 in Supplement 1. The CT group demonstrated significantly higher rates of coagulopathy (34.2% [69 of 202 patients] vs 14.2% [20 of 141 patients]), blood transfusions (83.9% [172 of 205 patients] vs 68.0% [100 of 147 patients]), and UAE (44.4% [91 of 205 patients] vs 9.5% [14 of 148 patients]) compared with the non-CT group.

Of the 205 patients who underwent dynamic CT scans, images were not available for 11 cases, and 14 cases did not meet the inclusion criteria because of factors such as uterine rupture. Consequently, 180 cases were included in the final analysis (Figure 2). We then analyzed the clinical characteristics of the PRACE and non-PRACE groups among the 180 patients who underwent dynamic CT imaging (Table 1). No significant differences were observed in maternal background and mode of delivery between the 2 groups. However, the PRACE group exhibited a notably higher total blood loss compared with the non-PRACE group (median [IQR], 3455 [2000-5070] mL in 54 cases vs 2500 [1500-2650] in 112 cases). Moreover, a higher percentage of patients in the PRACE group had minimum fibrinogen levels less than 150 mg/dL compared with the non-PRACE group (57.9% [33 of 57 patients] vs 23.3% [28 of 120 patients]). The PRACE group also received a significantly greater number of transfused RBCs (median [IQR], 10 [8-20] units vs 6 [2-10] units in the non-PRACE group) and fresh frozen plasma (median [IQR], 8 [6-19] units vs 4 [0-9] units in the non-PRACE group). Furthermore, fibrinogen was administered to a higher proportion of patients in the PRACE group compared with the non-PRACE group (43.1% [25 of 58 patients] vs 17.2% [21 of 122 patients]). In addition, UAE was performed significantly more frequently in the PRACE group compared with the non-PRACE group (86.2% [50 of 58 patients] vs 28.7% [35 of 122 patients]).

**Table 1.** Clinical Characteristics of Postpartum Hemorrhage by PRACE Status

Characteristic	Patients, No. (%)		*P* value
	PRACE negative (n = 122)	PRACE positive (n = 58)	
Clinical demographics			
Maternal age, median (IQR), y	33.0 (30.0-36.3)	33.0 (29.0-37.3)	.48
Primipara	78 (63.9)	41 (70.7)	.37
Conception through in vitro fertilization^a^	48/111 (43.2)	19/51 (37.3)	.47
Fibroids or adenomyosis^a^	12/120 (10.0)	5/57 (8.8)	.80
Gestational age of delivery, median (IQR), wk^a^	39.0 (38.0-40.0)^b^	39.0 (38.0-40.0)^c^	.82
Mode of delivery			
Labor analgesia^a^	16/120 (13.3)	14/57 (24.6)	.06
Labor induction or augmentation^a^	50/118 (42.3)	25/56 (44.6)	.78
Operative vaginal delivery	27 (22.1)	12 (20.7)	.83
Cesarean delivery	36 (29.5)	20 (34.4)	.50
Amount of blood loss, median (IQR), mL			
Pretransport^a^	2000 (1500-2650)^d^	2231 (1554-3000)^e^	.12
Total^a^	2500 (1716-3360)^d^	3455 (2000-5070)^f^	.005
Clinical status			
Maximum shock index ≥1.0^a^	61/120 (50.8)	31/57 (54.4)	.66
Minimum levels of hemoglobin, median (IQR), g/dL^a^	7.0 (6.2-8.1)^g^	6.5 (5.3-7.3)^c^	.002
Minimum fibrinogen levels <150 mg/dL^a^	28/120 (23.3)	33/57 (57.9)	<.001
Blood transfusion, median (IQR), units	92 (75.4)	56 (96.6)	<.001
Red blood cells	6 (2-10)	10 (8-20)	<.001
Fresh frozen plasma	4 (0-9)	8 (6-19)	<.001
Treatment			
Administration of fibrinogen	21 (17.2)	25 (43.1)	<.001
Administration of tranexamic acid	34 (27.9)	23 (39.7)	.11
Balloon tamponade	49 (40.1)	25 (43.1)	.71
Uterine artery embolization	35 (28.7)	50 (86.2)	<.001
Hysterectomy	2 (1.6)	2 (3.4)	.60
Maternal death	0	0	NA

Abbreviations: NA, not applicable; PRACE, postpartum hemorrhage resistant to treatment showing arterial contrast extravasation on dynamic computed tomography.

^a^ For variables with missing data, the denominator represents the number of cases for which data are available. Percentages are calculated on the basis of the available cases.

^b^ Data are available for 118 patients.

^c^ Data are available for 57 patients.

^d^ Data are available for 112 patients.

^e^ Data are available for 48 patients.

^f^ Data are available for 54 patients.

^g^ Data are available for 120 patients.

A univariable analysis was conducted to investigate risk factors associated with UAE and severe coagulopathy (defined as fibrinogen concentration <150 mg/dL). For UAE, potential risk factors included minimum fibrinogen levels less than 150 mg/dL (43.9% [36 of 82 patients] vs 26.3% [25 of 95 patients]) and the underlying cause of PPH (Table 2). For severe coagulopathy, candidate risk factors included maternal age 35 years or older (47.5% [29 of 61 patients] vs 35.3% [41 of 116 patients]), cesarean delivery (42.6% [26 of 61 patients] vs 25.9% [30 of 116 patients]), and the types of PPH (eTable 3 in Supplement 1).

**Table 2.** Univariable Analysis of Risk Factors for UAE in Dynamic Computed Tomography–Scanned Cases

Variable	Patients, No. (%)		*P* value
	UAE negative (n = 95)	UAE positive (n = 85)	
Maternal age ≥35 y	36 (37.9)	36 (42.4)	.54
Primipara	61 (64.2)	58 (68.2)	.57
Conception through in vitro fertilization^a^	35/86 (40.7)	32/76 (42.1)	.86
Fibroids or adenomyosis^a^	10/93 (10.8)	7/84 (8.3)	.59
Labor analgesia^a^	14/94 (14.9)	16/83 (19.3)	.44
Labor induction or augmentation^a^	39/92 (42.4)	36/82 (43.9)	.84
Operative vaginal delivery	23 (24.2)	16 (18.8)	.38
Cesarean delivery	30 (31.6)	26 (30.6)	.89
Minimum fibrinogen levels <150 mg/dL^a^	25/95 (26.3)	36/82 (43.9)	.02
Postpartum hemorrhage types			
Atonic uterus	65 (68.4)	17 (20.0)	<.001
Tissue	22 (23.2)	18 (21.2)	
PRACE	8 (8.4)	50 (58.8)	

Abbreviations: PRACE, postpartum hemorrhage resistant to treatment showing arterial contrast extravasation on dynamic computed tomography; UAE, uterine artery embolization.

^a^ For variables with missing data, the denominator represents the number of cases for which data are available. Percentages are calculated on the basis of the available cases.

In examining the association between risk factors and the severity of PPH (Table 3), PRACE emerged as the primary factor associated with the need for UAE (OR, 27.74, 95% CI, 10.52-83.14, with atonic uterus as the reference). Following this, tissue as the cause of PPH (OR, 3.67; 95% CI, 1.53-9.07, with atonic uterus as the reference) was identified as a factor significantly associated with UAE. However, maternal age, fibroid or adenomyosis, labor analgesia, labor induction or augmentation, cesarean delivery, and severe coagulopathy (defined as fibrinogen concentration <150 mg/dL) were not associated with the need for UAE. In addition, factors associated with risk of severe coagulopathy included PRACE (OR, 4.92; 95% CI, 2.23-11.33, with atonic uterus as the reference), labor induction or augmentation (OR, 2.42; 95% CI, 1.05-5.81, with spontaneous labor as the reference), and cesarean delivery (OR, 2.32; 95% CI, 1.02-5.40, with vaginal delivery as the reference).

**Table 3.** Multivariable Logistic Regression Analysis for Severity of PPH

Variable	OR (95% CI)	*P* value
Uterine artery embolization		
Maternal age ≥35 y	1.03 (0.45-2.31)	.95
Fibroids or adenomyosis	0.54 (0.13-2.03)	.37
Labor analgesia	0.71 (0.22-2.21)	.55
Labor induction or augmentation	1.43 (0.60-3.46)	.42
Cesarean delivery	1.18 (0.48-2.88)	.71
Minimum fibrinogen levels <150 mg/dL	0.92 (0.37-2.20)	.85
PPH types		
Atonic uterus	1 [Reference]	
Tissue	3.67 (1.53-9.07)	.004
PRACE	27.74 (10.52-83.14)	<.001
Minimum fibrinogen levels <150 mg/dL		
Maternal age ≥35 y	1.78 (0.84-3.77)	.13
Fibroids or adenomyosis	0.25 (0.05-1.01)	.07
Labor analgesia	0.60 (0.21-1.63)	.33
Labor induction or augmentation	2.42 (1.05-5.81)	.04
Cesarean delivery	2.32 (1.02-5.40)	.04
PPH types		
Atonic uterus	1 [Reference]	
Tissue	0.84 (0.29-2.25)	.73
PRACE	4.92 (2.23-11.33)	<.001

Abbreviations: OR, odds ratio; PPH, postpartum hemorrhage; PRACE, postpartum hemorrhage resistant to treatment showing arterial contrast extravasation on dynamic computed tomography.

## Discussion

This nationwide case-control study of severe PPH management across tertiary centers has yielded several crucial findings. Most notably, we observed that more than one-half of the severe PPH cases underwent CT imaging, with approximately one-third revealing PRACE. Our results demonstrate that PRACE is not merely a common occurrence but serves as a critical marker for severe PPH, evidenced by its associations with massive transfusion requirements, coagulopathy development, and the need for UAE. These findings position PRACE as a central determinant in severe PPH management strategies.

The cornerstone of effective PPH management lies in prompt and accurate diagnosis, particularly for cases that prove resistant to conventional interventions, such as uterotonics, balloon tamponade, or coagulopathy correction.^3,13^ Historically, dynamic CT has been underutilized in obstetric settings, largely because of the prevailing assumption that obstetric uterine bleeding manifests primarily as diffuse hemorrhage within the uterine cavity. Our findings challenge this long-held perspective, revealing that PRACE—characterized by focal arterial bleeding into the uterine cavity—represents a substantial proportion of cases traditionally attributed to atonic uterus or retained placental tissue.

The distinct clinical profile of PRACE emphasizes the necessity of incorporating advanced imaging techniques into standard PPH management protocols. Our multivariable analysis revealed that PRACE carries a remarkably high OR (27.74; 95% CI, 10.52-83.14) for UAE necessity. This high OR not only validates previous single-center findings but also establishes PRACE as a variable significantly associated with life-threatening complications.^4,5,6,13^ Notably, the absence of a direct correlation between coagulopathy and UAE suggests distinct pathophysiological mechanisms requiring different management approaches.

Our findings suggest the need to revise the traditional *4Ts* (tone, trauma, tissue, and thrombin) classification system^3,13^ to incorporate imaging findings. This integration would create a more comprehensive framework for PPH stratification, potentially leading to more targeted and effective interventions. The early identification of PRACE could enable clinicians to expedite interventional procedures such as UAE, potentially reducing both mortality and morbidity rates. Our study provides the foundational evidence needed to develop novel clinical algorithms that integrate imaging results with conventional clinical assessments, enabling more personalized and effective treatment strategies.

### Strengths and Limitations

This study’s primary strengths lie in its comprehensive scope, encompassing 352 severe PPH cases from 43 institutions, with dynamic CT evaluation in 180 cases—representing the largest investigation of its kind to date. However, several limitations warrant careful consideration.

First, the retrospective design introduces potential variability in treatment approaches across centers, despite presumed adherence to national guidelines.^14^ In addition, selection bias could have influenced which patients underwent CT imaging, as the decision was left to each institution’s discretion in the absence of a standardized protocol. Although CT imaging was performed in 58% of cases, highlighting its substantial clinical adoption in Japan, heterogeneity in its use may have affected case selection and subsequent interventions.

Second, PRACE was not an established concept during the study period, and its recognition varied across institutions. Treating physicians were not blinded to CT results; however, given the lack of standardized diagnostic criteria and management strategies at that time, PRACE findings alone were unlikely to have directly dictated UAE decisions. Therefore, the potential risk of bias from nonblinding was considered limited.

Third, the timing of CT imaging varied among institutions and physicians owing to the absence of standardized protocols. Although we previously proposed an algorithm recommending dynamic CT after initial resuscitation and failure of intrauterine balloon tamponade,^6^ its implementation was not uniform in clinical practice. This variability may have influenced intervention strategies and outcomes. Future prospective studies are essential to determine optimal timing for CT imaging and its impact on outcomes such as transfusion requirements and blood loss.

Fourth, access to dynamic CT imaging may be constrained in resource-limited settings, potentially limiting the generalizability of our findings. Differences in imaging availability across institutions and regions should be considered when applying these results to other settings.

## Conclusions

In this case-control study examining arterial contrast extravasation on dynamic CT in patients with severe PPH, we found that PRACE plays a critical role in guiding interventional decisions. Our findings indicate that dynamic CT imaging should be considered an essential component of the diagnostic algorithm for severe PPH, particularly in cases resistant to conventional treatment. Future prospective studies focusing on standardization of imaging protocols and their integration into clinical decision-making pathways will be crucial for optimizing maternal outcomes in severe PPH cases.

## Notes

Supplement 1. **eTable 1.** Clinical characteristics of the study population **eTable 2.** Clinical characteristics of PPH patients with and without CT imaging **eTable 3.** Univariable analysis of risk factors for severe coagulopathy in dynamic CT-scanned cases

Supplement 2.
Data Sharing Statement
