# Supplementary material for: Dynamic Computed Tomography Findings as Indicators of Uterine Artery Embolization in Postpartum Hemorrhage
Source: JAMA Netw Open. 2025 May 23;8(5):e2512209. doi: 10.1001/jamanetworkopen.2025.12209 (PMC12102700; doi:10.1001/jamanetworkopen.2025.12209)
Supplement: Supplement 1. — eTable 1. Clinical characteristics of the study population eTable 2. Clinical characteristics of PPH patients with and without CT imaging eTable 3. Univariable analysis of risk factors for severe coagulopathy in dynamic CT-scanned cases [file jamanetwopen-e2512209-s001.pdf]

Supplemental Online Content

Yamaguchi M, Sagara A, Nagayama Y, et al. Dynamic computed tomography findings as indicators of uterine artery embolization in postpartum hemorrhage. *JAMA Netw Open*. 2025;8(5):e2512209. doi:10.1001/jamanetworkopen.2025.12209

eTable 1. Clinical characteristics of the study population
eTable 2. Clinical characteristics of PPH patients with and without CT imaging
eTable 3. Univariable analysis of risk factors for severe coagulopathy in dynamic CT-scanned cases

This supplemental material has been provided by the authors to give readers additional information about their work.

© 2025 Yamaguchi M et al. *JAMA Network Open*.

eTable 1. Clinical characteristics of the study population

Clinical demographics	Total (n=352)
Maternal age, years	33.0 [30.0, 37.0]
Primipara	211 (60.0)
Conception through IVF*	120/317 (37.9)
Fibroids or adenomyosis*	27/341 (7.9)
Gestational age of delivery, weeks*	39.0 [38.0, 40.0]†
Mode of delivery	
Labor analgesia *	57/349 (16.3)
Labor induction or augmentation*	154/340 (45.3)
Operative vaginal delivery	78 (22.2)
Cesarean delivery	93 (26.4)
PPH types according to the clinical diagnosis at each institution	
Atonic uterus	258 (73.3)
Tissue	94 (26.7)
Amount of blood loss	
Pre-transport, mL*	1,961 [1,300, 2,613] ‡
Total, mL*	2,487 [1,637, 3,420] §
Clinical status	
Maximum shock index ≧ 1.0*	180/346 (52.6)
Minimum levels of hemoglobin, g/dL*	6.8 [5.9, 7.9]¶
Minimum fibrinogen levels <150 mg/dL*	89/343 (25.9)
Blood transfusion	272 (77.3)
RBC, units	6 [2, 10]
FFP, units	4 [0, 8]
Treatment	
Administration of fibrinogen	66 (18.8)
Administration of tranexamic acid	96 (27.3)
Balloon tamponade	144 (40.9)
Uterine artery embolization	105 (29.8)
Hysterectomy	8 (2.3)
Maternal death	1 (0.3)

Data are presented as median [Q1, Q3].

© 2025 Yamaguchi M et al. *JAMA Network Open*.

IVF: in-vitro fertilization, PPH: postpartum hemorrhage, RBC: red blood cells, FFP: fresh frozen plasma, CT: computed tomography, PRACE: postpartum hemorrhage resistant to treatment showing arterial contrast extravasation on dynamic CT.

* For variables with missing data, the denominator represents the number of cases for which data are available. Percentages are calculated based on the available cases. [†] Data available for 346, [‡] 308, [§] 327, and [¶] 349 patients.

© 2025 Yamaguchi M et al. *JAMA Network Open*.

eTable 2. Clinical characteristics of PPH patients with and without CT imaging

Variable	CT (−) n=147	CT (+) n=205	P value
Clinical demographics			
Maternal age, years	34.0 [30.0, 37.0]	33.0 [30.0, 37.0]	0.17
Primipara	75 (51.0)	136 (66.3)	0.003
Conception through IVF*	45/132 (34.1)	75/185 (40.5)	0.24
Fibroids or adenomyosis*	10/139 (7.2)	17/202 (8.4)	0.68
Gestational age of delivery, weeks*	39.0 [38.0, 40.0]†	39.0 [38.0, 40.0]‡	0.45
Mode of delivery			
Labor analgesia *	19/147 (12.9)	38/202 (18.8)	0.14
Labor induction or augmentation*	66/142 (46.5)	88/198 (44.4)	0.71
Operative vaginal delivery	31 (21.1)	47 (22.9)	0.68
Cesarean delivery	30 (20.4)	63 (30.7)	0.03
PPH types			
Atonic uterus	101 (68.7)	157 (76.6)	0.10
Tissue	46 (31.3)	48 (23.4)	0.10
Amount of blood loss			
Pre-transport, mL*	1,800 [1,200, 2,498] §	2,000 [1,500, 2,863] ¶	0.01
Total, mL*	2,110 [1,507, 3,108] #	2,600 [1,885, 3,700] ‖	0.003
Maximum shock index ≧ 1.0*	75/144 (52.1)	105/200 (52.5)	0.94
Minimum levels of hemoglobin, g/dL*	6.8 [6.0, 8.3] ††	6.8 [5.9, 7.8] ‡‡	0.32
Minimum fibrinogen levels <150 mg/dL*	20/141 (14.2)	69/202 (34.2)	＜0.001
Blood transfusion	100 (68.0)	172 (83.9)	＜0.001
RBC, units	4 [0, 8]	6 [4, 12]	＜0.001
FFP, units	0 [0, 6]	6 [0, 12]	＜0.001
Treatment			
Administration of fibrinogen	17 (11.6)	49 (23.9)	0.003
Administration of tranexamic acid	34 (23.1)	62 (30.2)	0.15
Balloon tamponade	60 (40.8)	84 (41.0)	0.98
Uterine artery embolization	14 (9.5)	91 (44.4)	＜0.001
Hysterectomy	3 (2.0)	5 (2.4)	0.80

© 2025 Yamaguchi M et al. *JAMA Network Open*.

Maternal death	1 (0.7)	0 (0)	0.24

Data are presented as median [Q1, Q3].

IVF: in-vitro fertilization, PPH: postpartum hemorrhage, RBC: red blood cells, FFP: fresh frozen plasma, CT: computed tomography, PRACE: postpartum hemorrhage resistant to treatment showing arterial contrast extravasation on dynamic CT.

* For variables with missing data, the denominator represents the number of cases for which data are available. Percentages are calculated based on the available cases. [†] Data available for 146, [‡] 200, [§] 128,[¶] 100, [#] 140, [‖] 187, [††] 147, and [‡‡] 202 patients.

© 2025 Yamaguchi M et al. *JAMA Network Open*.

eTable 3. Univariable analysis of risk factors for severe coagulopathy in dynamic CT-scanned cases

Variable	Minimum fibrinogen levels, mg/dL		P value
	≥150 (n=116)	<150 (n=61)	
Maternal age ≥ 35 years	41(35.3)	29 (47.5)	0.11
Primipara	75(64.7)	42 (68.9)	0.58
Conception through IVF*	44/107 (41.1)	23/52 (44.2)	0.71
Fibroids or adenomyosis*	13/116 (11.2)	3/58 (5.2)	0.17
Labor analgesia *	19/115 (16.5)	11/59 (18.6)	0.73
Labor induction or augmentation*	46/111 (41.4)	29/60 (48.3)	0.39
Operative vaginal delivery	25 (21.6)	13 (21.3)	0.97
Cesarean delivery	30 (25.9)	26 (42.6)	0.02
PPH types			<0.001
Atonic uterus	60	20	
Tissue	32	8	
PRACE	24	33	

Data are presented as n (%).

IVF: in-vitro fertilization, PPH: postpartum hemorrhage, PRACE: postpartum hemorrhage resistant to treatment showing arterial contrast extravasation on dynamic CT, RBC: red blood cells, FFP: fresh frozen plasma, UAE: uterine artery embolization.

* For variables with missing data, the denominator represents the number of cases for which data are available. Percentages are calculated based on the available cases.

© 2025 Yamaguchi M et al. *JAMA Network Open*.
