# Supplementary material for: Dynamic Computed Tomography Findings as Indicators of Uterine Artery Embolization in Postpartum Hemorrhage
Source: JAMA Netw Open. 2025 May 23;8(5):e2512209. doi: 10.1001/jamanetworkopen.2025.12209 (PMC12102700; doi:10.1001/jamanetworkopen.2025.12209)
Supplement: Supplement 2. — Data Sharing Statement [file jamanetwopen-e2512209-s002.pdf]

Data Sharing Statement

Yamaguchi. Dynamic Computed Tomography Findings as Indicators of Uterine Artery Embolization in Postpartum Hemorrhage. *JAMA Netw Open*. Published May 23, 2025. doi:10.1001/jamanetworkopen.2025.12209

Data
Data available: No
